# *TNF* gene polymorphisms in cystic fibrosis patients: contribution to the disease progression

**DOI:** 10.1186/1479-5876-11-19

**Published:** 2013-01-23

**Authors:** Galina Shmarina, Alexander Pukhalsky, Nika Petrova, Ekaterina Zakharova, Lucine Avakian, Nikolai Kapranov, Vladimir Alioshkin

**Affiliations:** 1 Department of Cystic Fibrosis, Research Centre for Medical Genetics, 1 Moskvorechie Street, Moscow, 115478, Russia; 2 Laboratory of Cytokines, G. N. Gabrichevsky Institute of Epidemiology and Microbiology, Moscow, Russia; 3 Laboratory of Genetic Epidemiology, Research Centre for Medical Genetics, Moscow, Russia; 4 Laboratory of Metabolic Diseases, Research Centre for Medical, Moscow, Russia; 5 G. N. Gabrichevsky Institute of Epidemiology and Microbiology, Moscow, Russia

**Keywords:** TNF, Gene polymorphism, Cystic fibrosis, Inflammation, Liver disease, Osteoporosis, Tuberculosis, Asthma

## Abstract

**Background:**

It is well known that the disease progression in cystic fibrosis (CF) patients may be diverse in subjects with identical mutation in CFTR gene. It is quite possible that such heterogeneity is associated with *TNF*-*α* and/or *LT*-*α* gene polymorphisms since their products play a key role in inflammation. The aim of the study was to investigate the possible roles of *TNF* gene polymorphisms in CF disease phenotype and progression.

**Methods:**

198 CF patients and 130 control subjects were genotyped for both *TNF*-*α*–*308GA* and *LT*-*α* + *252AG* polymorphisms.

**Results:**

The carriers of the *TNF*-*α*–*308A* allele more frequently had asthma as compared to patients homozygous for the *TNF*-*α*–*308 G* allele. In 9 of 108 (8.3%) of *LTα* + *252AA* carriers, tuberculosis infection has been documented, whereas there was no case of tuberculosis among patients, either homozygous or heterozygous for *LTα* +*252 G* alleles (*p* = 0.01). We never observed virus hepatitis among *LTα* + *252GA* carriers. The genotypes *TNF*-*α*–*308GG* – *LT*-*α* + *252AA* and *TNF*-*α*–*308GA* – *LT*-*α* + *252AG* were unfavorable with regard to liver disease development (both *p* < 0.05). It was also shown that neutrophil elastase activity was higher in sputum specimens from high TNF producers with genotypes *TNF*-*α*–*308GA* or *LT*-*α* + *252GG*. In addition the carriers of such genotypes demonstrated a higher risk of osteoporosis development (*p* values were 0.011 and 0.017, respectively).

**Conclusions:**

The carriers of genotypes, which are associated with higher TNF-α production, demonstrated increased frequency of asthma, higher levels of neutrophil elastase, and decrease of bone density. On the contrary, the carriers of genotypes associated with low TNF-α production showed a higher frequency of tuberculosis infection.

## Background

The Major Histocompatibility Complex (MHC) contains genes essential to both the adaptive and innate immune systems. In humans, these genes are referred to as HLA genes. Genes within the MHC traditionally divided into three different subregions. Class I and II regions contain genes encoded molecules that are responsible for antigen presentation to T cells. The human Class III region is the most gene-dense and highly conserved region of the human genome [1]. Within this region *TNFα* and *TNF*-β (*LTα*) genes are located close to each other [2]. The gene products tumor necrosis factor (TNF)-α and TNF-β, also known as lymphotoxin-α (LT-α), exhibit a broad spectrum of inflammatory and immunomodulatory activities. In particular, at the level of hypothalamus TNF-α stimulates hypothalamic-pituitary-adrenal axis, in the liver it stimulates acute phase response and increases insulin resistance in different tissues. At the level of macrophages it stimulates phagocytosis and the production of PGE2. In addition, TNF-α is a potent chemoattractant, which helps neutrophils to stick to the endothelial cells for migration. The effects of LT-α are similar substantially to

TNF-α, but LT-α is also important for the development of lymphoid organs [3-5]. It is obvious that both cytokines play an important role in pathogenesis of many inflammatory disorders including cystic fibrosis (CF), the most common autosomal recessive disease in Caucasian population. Although there is no doubt that etiology of CF is directly associated with the mutation in the *cystic fibrosis transmembrane conductance regulator* (*CFTR*) gene, it is very difficult to connect the disease clinical course with the type of mutation. Indeed, it is well known that CF progression may be diverse among either siblings or unrelated patients with identical mutation in *CFTR* gene. Apparently there are other factors including genetic ones, which may determine the course of disease [6,7]. It is quite possible that such heterogeneity is associated with *TNFα* and/or *LTα* gene polymorphisms. A polymorphism in the promoter region of the

*TNFα* gene at nucleotide −308, relative to the transcription start site, may be important in determining the host TNF-α response [8]. There are two alleles at the polymorphic site, *TNFα308 G* and *TNFα308A*. In normal population *TNFα308 G* homozygosity is the predominant genotype. The *TNFα308GA* polymorphism has a small, but significant, functional effect, with the A allele being associated with higher constitutive and inducible levels of transcription for TNF-α than the G allele [9]. Within the first intron of the *LTα* gene at position +252, there is *Nco*I restriction polymorphism, consisting of a Guanine (*LTα* + *252 G*) on one allele and an Adenine (*LTα* + *252Α*) on the alternate allele. The presence of G at this position defines the allele which is less frequent one in white subjects and is associated with higher TNF-α and TNF-β production [10,11]. Recently it has been shown that lung function is significantly lower in CF patients with *TNFα308A* polymorphism [12]. It has been also demonstrated that certain *TNFα* polymorphisms, other than −308 G/A polymorphic loci, are also associated with severity of CF lung disease in Czech and Belgian patients [13].

The aim of the study was to investigate the possible roles of *TNF* gene polymorphisms in CF disease phenotype and progression. To address this issue, we genotyped 198 CF patients and 130 control subjects for both *TNF*-*α*–*308GA* and *LT*-*α* + *252AG* polymorphisms.

## Methods

### Patients

A total of 198 patients (mean age, 12.7 ± 0.6 years) from the Cystic Fibrosis Department of the Research Centre for Medical Genetics (Moscow) were enrolled into the study. CF was diagnosed by increased chloride concentrations (>60 mmol/l) in a sweat test, typical clinical symptoms of the disease, and/or detection of mutations in both *CFTR* alleles. Clinical, biological and functional data were obtained from hospital records from the previous 2 to 15 years. The data included date of birth, sex, *CFTR* genotype, pulmonary function tests, nutritional status, airways microbiology, CF and non-CF complications (cirrhosis, osteoporosis, pulmonary aspergillosis, asthma, tuberculosis, virus hepatitis, etc.). Lung function was assessed by spirometry in children >4 years during periods of clinical stability. Respiratory microbial flora was determined by microscopy and culture of lower respiratory tract secretions or throat swabs realized every routine visit to the CF Department. Chronic airway colonization with *Pseudomonas aeruginosa* was defined by the persistence of the pathogen in at least three airway samples for at least 6 months. 127 individuals were chronically colonized with the mucoid form of *P*. *aeruginosa*. The *CFTR* genotype in 138 CF patients was homozygous or heterozygous for F508del (ΔF508). Forced expiratory volume in 1 sec (FEV1) and forced vital capacity (FVC) values averaged 78.1 ± 4.4 and 70.1 ± 4.0% predicted, respectively. Characteristics of patient groups with different *TNF* gene polymorphisms are presented in Additional file 1: Table S1 and Additional file 2: Table S2. The patients were treated with basic therapy (mucolytics, multivitamins, high calorie diet, microspheric enzymes). Some individuals received anti-inflammatory therapy including azithromycin, nimesulide or/and prednisolone in low doses. In the case of acute pulmonary exacerbation, antibacterial treatment depended on the microbiology analysis of the sputum. Individuals with *P*. *aeruginosa* infection were treated by cephalosporins of third generation in combination with aminoglycosids or ciprofloxacin.

### Blood collection and sputum processing

Blood was collected in tubes with heparin (25 IU/ml) by venipuncture. The sputum samples were placed into the container with ice and delivered to the laboratory within 1 h. The weight of each sputum sample was calculated. The same weight of phosphate-buffered saline without Ca2+ and Mg2+ was added to the sputum sample. The mixture placed on vortex for 10 sec and then on the rocker for 30 min. The sample was filtered through a 100 μm filter to remove the mucus. The filtrate has been centrifuged at 400 × g for 10 min at 4°C to pellet the cells. The supernatant has been harvested, aliquoted and stored at −60°C. Protein concentrations in the samples were measured by Bradford’s method.

### Genotyping

Genomic DNA was extracted from anti-coagulated blood by a conventional proteinase K digestion/phenol-chloroform extraction method. Typing of *TNFα* promoter gene polymorphism (rs1800629, –308 G/A) was performed by polymerase chain reaction (PCR) amplification (using a 5^′^primer 5^′^-AGGCAATAGGTTTTGAGGGCCAT3^′^ and 5^′^-TCCTCCCTGCTCCGATTCCG3^′^ as the 3^′^primer) and NcoI digestion as described by Zhang DL *et al*. [14]. PCR was carried out in 25-μL volume containing 0.5 μg of genomic DNA, 1 μM of each primer, 1.5 U of Taq DNA polymerase, 0.2 mM of each 2^′^-deoxiribonucleoside 5^′^-triphosphate, 67 mM Tris–HCl, pH 8.4, 2 mM MgCl2, 16.6 mM (NH4)SO4, and 20 μg/ml BSA. The cycling condition consisted of an initial activation of Taq polymerase at 94°C for 5 min followed by 35 cycles of denaturation at 94°C for 45 sec, annealing at 60°C for 45 sec, and extension at 72°C for 45 sec. The PCR products were digested with 8 U of *Nco* I at 37°C for 6 h. Digested DNA was analyzed on 8% polyacrylamide gels. Ethidium bromide staining of the gel demonstrated the original 107 basepairs fragment (homozygous patients for allele *TNFα308A*, lacking NcoI site), three fragments of 102, 87 and 20 basepairs (heterozygous patients), or two fragments of 87 and 20 basepairs of size (homozygous patients for the allele TNF-α–308 G). The *Nco* I polymorphism in intron 1 of the *LTα* (rs909253, +252 A/G) was determined by PCR-restriction fragment length polymorphism method. A 782 basepairs fragment of the LT-α genomic sequence, including the polymorphic NcoI site, was amplified with a sense primer (5^′^- CCGTGCTTCGTGCTTTGGACTA 3^′^) and an antisense primer (5^′^- AGAGGGGTGGATGCTTGGGTTC3^′^) [14,15]. PCR was carried out in 25-μL volume containing 0.5 μg of genomic DNA, 1 μM of each primer, 1.5 U of Taq DNA polymerase, 0.2 mM of each 2^′^-deoxiribonucleoside 5^′^-triphosphate, 67 mM Tris–HCl, pH 8.4, 2 mM MgCl2, 16.6 mM (NH4)SO4, and 20 μg/ml BSA. The cycling condition consisted of an initial activation of Taq polymerase at 94°C for 5 min followed by 35 cycles of denaturation at 94°C for 1 min, annealing at 50°C for 1 min, and extension at 72°C for 1 min. The PCR products were digested with 8 U of *Nco* I at 37°C for 6 h. Digested DNA was analyzed on 8% polyacrylamide gels. Ethidium bromide staining of the gel demonstrated the original 782 basepairs fragment (homozygous patients for allele *LTα* + *252A*), three fragments of 782, 586 and 196 basepairs (heterozygous patients), or two fragments of 586 and 196 basepairs of size (homozygous patients for the allele *LTα* + *252 G*).

### Assay of human leukocyte elastase activity

The method is based on the ability of neutrophil elastase to interact with specific chromogenic substrate *N*-methoxysuccinyl-ala-ala-pro-val *p*-nitro anilide (Sigma, St Louis, MO, USA), forming a colored complex with maximum of absorbance at 410 nm [16]. The standard assay was performed as described earlier [17]. Finally, the value of neutrophil elastase activity was normalized to the protein content in each sample of the sputum extract.

### Bone density assessment

Fifty four patients were undergone to bone mineral density (BMD, g/cm2) assessment. BMD was assessed at the lumbar spine (L1-L4) by dual energy x-ray absorptiometry using a Lunar Prodigy Bone Densitometer (GE Lunar Corporation, WI, USA). The results were expressed as Z score for age, sex and ethnicity according to the reference data given by the GE Lunar Corporation software. Z scores were calculated by subtracting the sex- and age-specific population mean BMD from the CF subject’s BMD; this value was then divided by the standard deviation (SD) of the sex and age-specific mean. Z scores were classified according to the standards recently proposed for pediatric subjects to define bone density reduction [18]. We considered normal BMD a lumbar spine Z score above −1, mild BMD reduction a lumbar spine Z score lower than −1.0 but higher than −2, severe BMD reduction a lumbar spine Z score lower than −2.0.

### Statistical analysis

The differences in allele/genotype frequencies between patients and controls were analyzed by the Fisher’s exact test. This test was also used for assessment of the differences in clinical course between patients with different genotypes. The levels of neutrophil elastase activity in sputa, the data of pulmonary function tests as well as z-score values were compared by unpaired Student’s *t*-test. *P* values less than 0.05 were considered significant.

### Ethics approval

The study was approved by the Ethics Committee of the Research Centre for Medical Genetics.

## Results

There were no significant differences between CF patients and healthy subjects for the *TNF* genotype frequencies (Table 1). In order to evaluate the contribution of individual polymorphisms in the disease progression, we pooled the findings about *TNF*-*α* and *LT*-*α* genes. In spite of such integration no significant differences between CF patients and general population of Moscow residents have been found (Additional file 3: Table S3). We also did not reveal any associations between TNF genotypes and the gender distribution (see Additional file 1: Table S1).

**Table 1:** *TNF* gene polymorphisms in cystic fibrosis patients and healthy children

**Genotype**	**Healthy children**	**CF patients**	***p***
*TNF*-*α*–*308GG*	93 (72%)	145 (73%)	0.728
*TNF*-*α*–*308GA*	33 (26%)	53 (27%)	0.913
*TNF*-*α*–*308AA*	3 (2%)	Not found	0.118
Allele frequency, G/A	0.85/0.15	0.87/0.13	0.921
*LT*-*α* + *252GG*	13 (10%)	12 (6%)	0.272
*LT*-*α* + *252GA*	42 (33%)	67 (36%)	0.633
*LT*-*α* + *252AA*	72 (57%)	110 (58%)	0.854
Allele frequency, A/G	0.27/0.73	0.24/0.76	0.263

### Associations with lung diseases

**Figure 1:**
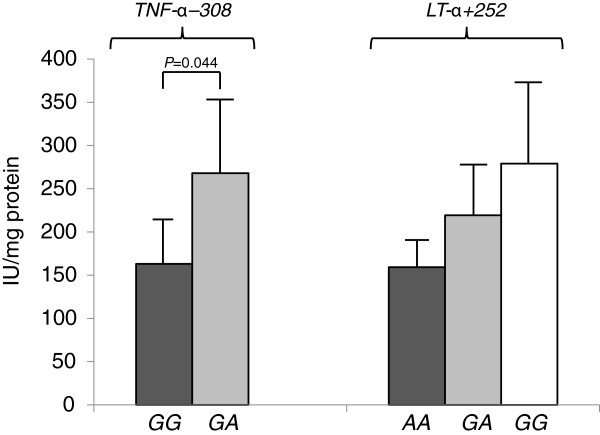
**Sputum elastase levels in carriers of different TNF**-**α** -**308 and LT**-**α** +**252 genotypes among cystic fibrosis patients.**

Followed the classical single polymorphism approach, we have found no association between individual *LT*-*α* or *TNF*-*α* single SNPs and lung function in our CF cohort (Additional file 2: Table S2) but neutrophil elastase activity was higher in sputum specimens from carriers of genotypes *TNF*-*α*–*308GA* or *LT*-*α* + *252GG*, associated with increased TNF-α production (Figure 1). In the same time the patients with genotype *TNF*-*α*-*308GA* – *LT*-*α* + *252AA* did have a better lung function compared to subjects from other *TNFα* – *LT*-*α* genotype groups (Table 2). The groups did not differ significantly in term their age (mean values ranged from 11.2 to 13.1 years) or *P*. *aeruginosa* infection (from 58.3 to 75% patients in the groups were chronically colonized with the pathogen).

**Table 2:** Contribution of individual *TNF*-*α* and *LT*-*α* gene polymorphisms in CF lung disease progression

***TNF *genes**	**FVC**	p*	**FEV**_**1**_	p*
***TNF***-***α***-***308GA*** – ***LT***-***α*** + ***252AA***	**96**.**9** ± **9**.**5** (**n** = **12**)		**91**.**5** ± **10**.**4** (**n** = **12**)	
*TNF*-*α*-*308GG* – *LT*-*α* + *252AA*	78.8 ± 2.6 (n = 94)	0.025	70.3 ± 2.8 (n = 94)	0.015
*TNF*-*α*-*308GA* – *LT*-*α* + *252AG*	72.5 ± 4.2 (n = 27)	0.008	66.4 ± 5.5 (n = 27)	0.009
*TNF*-*α*-*308GG* – *LT*-*α* + *252AG*	78.1 ± 3.8 (n = 33)	0.025	72.6 ± 4.6 (n = 32)	0.027
*TNF*-*α*-*308GG* – *LT*-*α* + *252GG together with* *TNF*-*α*-*308GA* – *LT*-*α* + *252GG*	76.1 ± 6.8 (n = 11)	0.029	69.3 ± 8.9 (n = 11)	0.039

Boldface shows the favorable genotype in term of CF lung disease progression.

FVC, forced vital capacity; FEV_1_, forced expiratory volume in 1 sec.

* comparison with *TNF*-*α*-*308GA* – *LT*-*α* + *252AA* genotype.

It was also shown that the carriers of the *TNFα308A* allele more frequently had asthma as compared to patients homozygous for the *TNFα308 G* allele (Table 3). These data are in line with the results of other studies including meta-analysis [19] suggesting a strong positive association between –308A allele of *TNFα* gene and asthma development. In Russian Federation the prevalence of asthma ranges from 2.2% to 7% in adults and 10% in children [20]. So, in theory, these proportions of CF pediatric patients would be expected to have concomitant asthma. In our CF cohort 7.5% of patients had asthma diagnosis.

**Table 3:** **Frequencies of concomitant diseases in CF patients with different *TNF***-***α *and *LT***-***α *gene polymorphisms**

**Genotype**	**Asthma**	**Tuberculosis**	**Virus hepatitis**
*TNF*-*α*–*308 GG*	5.8%	5.0%	6.3%
(n = 145)	(8/145)	(7/140)	(8/143)
*TNF*-*α*–*308GA*	14.0%	2.9%	3.8%
(n = 53)	(7/53)	(2/51)	(2/53)
*P*-value	0.068	0.94	0.9
*LT*-*α* + *252AA*	6.5%	8.3%	6.4%
(n = 110)	(7/110)	(9/108)	(7/109)
*LT*-*α* + *252GA*	7.8%	0%	0%
(n = 67)	(5/67)	(0/65)	(0/65)
*LT*-*α* + *252GG*	10.0%	0%	16.7%
(n = 12)	(1/12)	(0/11)	(1/12)
*P* value		P_A/A,G/A_ = 0.013	P_A/A,G/A_ = 0.035
		P_A/A,G/G+G/A_ = 0.007	P_A/A,G/G+G/A_ = 0.088

We did not find any association between *TNFα308* polymorphism and tuberculosis susceptibility (see Table 3). However, in 9 of 108 (8.3%) of *LTα* + *252AA* carriers tuberculosis infection has been documented. At the same time there was no case of tuberculosis among patients, both homozygous and heterozygous for *LTα* + *252 G* alleles (*p* = 0.01) (see Table 3). The results are similar to the recent data of García-Elorriaga *et al*. showing that healthy subjects have had significantly high frequency of the *LTα* + *252A* allele compared to groups of tuberculosis patients [21].

### Associations with CF related liver diseases

Liver disease is the second leading cause of death in patients with CF [22]. It is estimated that 40% of CF patients develop liver disease, characterized by focal portal fibrosis and cholestasis, but only 1-5% of these cases progress to portal hypertension and end-stage liver disease [23,24]. In our study 14 of 198 patients (7.1%) had severe cirrhosis with portal hypertension. There were no significant association of the cirrhosis development with both TNF-α–308 G/A or LT-α + 252A/G gene polymorphisms. In the same time, as can be seen in Table 4, genotypes *TNFα308GG* – *LTα* + *252AA* and *TNFα308GA* – *LTα* + *252AG* were unfavorable with regard to liver disease but appeared to have little if any effect on susceptibility to portal hypertension development. Thus, 25.8% patients exhibiting genotypes *TNFα308GA* – *LTα* + *252AG* and 31% patients having *TNFα308GG* – *LTα* + *252AA* demonstrated ultrasound signs of cirrhosis development whereas the frequencies of portal hypertension in these two groups were 6.2% and 13.8%, respectively. The data did not differ from those in CF patients with other *TNF* genotypes (Additional file 4: Table S4). Similarly, Suneetha et al. have shown that the combination *TNFα308GG* – *LTα* + *252AA* was more common among HBV infected patients with severe liver disease than those with mild disease on the basis of histological activity index and fibrosis score [25]. In our CF cohort 9 of 198 (4.5%) patients were chronically infected with hepatitis viruses (5 patients - with HCV and 4 subjects - with HBV). Among them there was only one patient who developed severe liver disease with portal hypertension. As can be seen in Table 3, *LTα* + *252A*/*G* gene polymorphism (but not *TNFα308 G*/*A*) was associated with susceptibility to virus hepatitis in our CF patients. Indeed, among *LTα* + *252 G* carriers the patients with virus hepatitis have not been found, and only patient with *LTα* + *252 G*/*G* genotype were infected with HCV. Our data are in line with previous study by Goyal et. al. suggesting that *LTα* + *252AA* allele is significantly more common in non-CF subjects infected with HCV as compared to healthy controls [26].

**Table 4:** **Contribution of individual *TNF***-***α *and *LT***-***α *gene polymorphisms in CF associated liver disease**

***TNF *genes**	**Cirrhosis (total)**	p*	p**	**Cirrhosis (without PH)**	p*	p**
*TNF*-*α*-*308GA*–*LT*-*α* + *252AA*	1/13 (7.7%)	0.061	0.018	0/13	0.028	0.016
***TNF***-***α***-***308 G***–***LT***- + ***252AA***	**31**/**97** (**32**%)	—	>**0**.**1**	**25**/**97** (**25**.**8**%)	—	>**0**.**1**
***TNF***-***α***-***308GA***–***LT***- + ***252AG***	**13**/**29** (**44**.**8**%)	>**0**.**1**	—	**9**/**29** (**31**.**0**%)	>**0**.**1**	—
*TNF*-*α*-*308GG*–*LT*- + *252AG*	5/37 (13.5%)	0.023	0.005	4/37 (10.8%)	0.045	0.041
*TNF*-*α*-*308GG*–*LT*- + *252GG together with*	2/12 (16.7%)	>0.1	0.087	0/12	0.036	0.029
*TNF*-*α*-*308GA*–*LT*- + *252GG*						

Boldface shows the unfavorable genotype in term of CF associated liver disease progression.

PH, portal hypertension.

* comparison with *TNF*-*α*-*308GG* – *LT*-*α* + *252AA*.

** comparison with *TNF*-*α*-*308GA* – *LT*-*α* + *252AG*.

### Associations with osteoporosis

**Figure 2:**
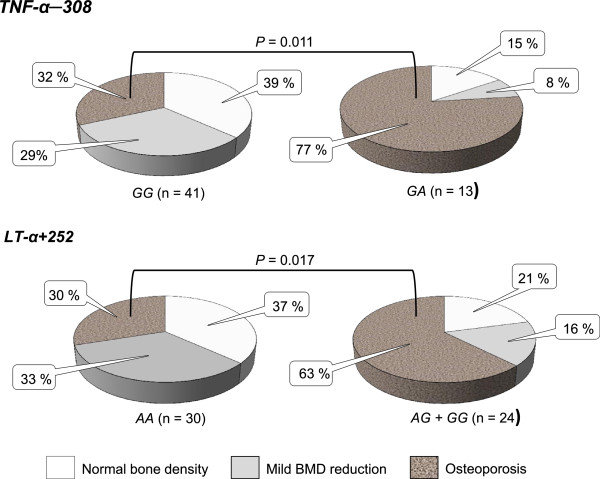
**Frequency of the bone mineral density decrease in carriers of different *TNF***-***α*** -***308 *and *LT***-***α*** +***252 *genotypes among cystic fibrosis patients.**

Mean lumbar spine BMD Z score in CF patients homozygous for the *TNF*-*α*–*308* G allele was significantly higher than that in CF carriers of the *TNF*-*α*–*308A* allele (−1.6 ± 0.3 *vs* −3.0 ± 0.5, *p* = 0.027). Bone status of the two groups is shown in Figure 2. Median age of patients was not statistically differed in these two groups. Sixteen of 41 patients (39.0%, 11 males and 5 females) homozygous for the *TNF*-*α*–*308* G allele had a normal BMD, with a Z score higher than −1. A mild BMD reduction (with lumbar spine Z score lower than −1.0, but higher than −2.0) was documented in 12 subjects (29.3%, 5 males and 7 females). A condition of severe BMD reduction, with a lumbar spine Z score less than −2, was evident in 13 patients (31.7%, 6 males and 7 females). At the same time 10 of 13 *TNF*-*α*–*308A* carriers (76.4%, 4 males and 6 females) demonstrated a severe BMD reduction. It was also shown the statistically significant differences in bone density among patients homozygous for *LT*-*α* + *252A* allele and *LT*-*α* + *252 G* carriers. The last were found to demonstrate decreased values of lumbar spine BMD Z score (−2.5 ± 0.4 *vs* – 1.5 ± 0.3, *p* = 0.048) and increased osteoporosis frequency. Thus, 15 of 24 CF patients (62.5%, 8 males and 7 females) with *LT*-*α* + *252 G* had severe BMD reduction. In this patient group a mild BMD reduction and normal BMD were found in 4 (16.7%) and 5 (20.8%) subjects, respectively. In contrast, most of 30 patients homozygous for *LT*-*α* + *252A* allele had normal BMD or mild BMD reduction (11 and 10 individuals, respectively). No statistical difference was found among the groups in terms of patient age.

## Discussion

Many studies have investigated the potential role of *TNF*-α and *LT*-α gene polymorphisms in the development of various diseases [19,21,26-30]. Several lines of evidence suggest that *TNF* genes may be implicated in the pathogenic mechanisms of inflammatory diseases. In case of CF we could say only about CF progression and development of the disease complications, but not about predisposition to suffer from CF, since the etiology of it is absolutely clear and associated with mutation in both CFTR alleles. It might be supposed that the influence of *TNF* gene polymorphisms upon the disease progression in CF patients is associated with high or low TNF production in the carriers of different *TNF* genotypes. Indeed, it was shown that allele *TNFα*

*308A* has been associated with higher inducible levels of gene transcription and TNF-α protein production [27,31]. Among the polymorphisms of *LT*-α gene *LTα* + *252 G* allele is associated with higher TNF-α and TNF-β production [10,11]. Clinical observations indirectly confirm the data received in *in vitro* experiments on human cell lines. Thus, the results of meta-analysis carried out to explore the association between the *TNFα* –308GA polymorphism and asthma development suggested that *TNFα* –308A allele may be a risk factor in the etiology of the disease. In the subgroup analysis by atopic status, significant elevated risks of asthma were associated with A allele carriers in atopic population [19]. It was also shown that the risk for the –308A allele in asthma was greater in females [28]. On the contrary the *TNFα308GG* genotype may have a protective role in asthma pathogenesis [29]. At the same time, highly conserved ancestral haplotype (AH) 8.1 including among others *TNFα308A* and *LTα* + *252 G* alleles, is an important genetic modifier of lung disease in CF. Although 8.1 AH is associated with delayed onset of respiratory colonization with *S*. *aureus* and *P*. *aeruginosa* in young CF patients. An elevated inflammatory response being beneficial in the early stages of childhood CF becomes destructive as chronic infection ensure in older patients [32]. On the contrary, primary biliary cirrhosis is associated with reduced carriage of the high production *TNFα308A* allele [30]. This is in keeping with a protective role of TNF-α against the disease. In whole, it is safe to say that TNF-α is a potent immunomediator and pro-inflammatory cytokine that has been implicated in the pathogenesis of a large number of human diseases.

Our data received in the cohort of CF patients confirm these observations. Thus, the carriers of genotypes, which are associated with higher TNF production, demonstrated more frequency of asthma, higher levels of neutrophil elastase, and decrease of bone density. At the same time, low TNF producers showed a higher frequency of tuberculosis infection. We believe that the CF complications associated with *TNF* gene polymorphisms are the same that *TNF* gene associated diseases in general population. Simply, there is a strong possibility that such genetically predisposed diseases will be diagnosed in CF subjects in time since most of them undergo a medical examination at least twice a year.

## Conclusions

The work shows that the carriers of genotypes, which are associated with higher TNF production, demonstrate more frequency of asthma, higher levels of neutrophil elastase, and decrease of bone density. On the contrary, the carriers of genotypes associated with low TNF production show a higher frequency of tuberculosis and virus hepatitis infection. Thereby, *TNF* genotyping may be useful for prognosis and for choice of therapeutic strategy in CF patients.

## Competing interests

The authors declare that they have no competing interests.

## Authors’ contributions

GS and AP made an equal contribution in data analysis and manuscript planning and writing. VA participated manuscript writing and approved the final version. NP and EZ were responsible for genotyping. LA conducted the clinical examination and performed data collection. NK revised the manuscript critically. All authors read and approved the final manuscript.

## Supplementary Material

Additional file 1 **Table S1.** Characteristics of the cystic fibrosis patients with different *TNF* gene polymorphisms. Click here for file

Additional file 2 **Table S2.** Forced vital capacity (FVC and FEV_1_) in CF patients with different TNF genotypes. Click here for file

Additional file 3 **Table S3.** Frequencies of various TNF genotypes among CF patients and Healthy subjects. Click here for file

Additional file 4 **Table S4.** Contribution of individual *TNF*-*α* and *LT*-*α* gene polymorphisms in CF associated cirrhosis with portal hypertension. Click here for file
