# Supplementary material for: TNF gene polymorphisms in cystic fibrosis patients: contribution to the disease progression
Source: J Transl Med. 2013 Jan 23;11:19. doi: 10.1186/1479-5876-11-19 (PMC3565881; doi:10.1186/1479-5876-11-19)
Supplement: Additional file 1 — Table S1. Characteristics of the cystic fibrosis patients with different TNF gene polymorphisms. [file 1479-5876-11-19-S1.docx]

**Table S1**  Characteristics of the cystic fibrosis patients with different *TNF* polymorphisms

|  | *TNF-α* gene polymorphisms | |  | *LT-α* gene  polymorphisms | | |
| --- | --- | --- | --- | --- | --- | --- |
|  | GG | GA |  | GG | GA | AA |
| Patient number | 145 | 53 |  | 12 | 67 | 110 |
| Sex (Male/Female) | 73/72 | 30/23 |  | 7/5 | 32/35 | 60/50 |
| Age (years) | 12.6±0.3 | 12.9±0.5 |  | 13.0±1.3 | 11.8 ±0.5 | 13.0 ± 0.4 |
| Weight (% predicted) | 82.3±2,7 | 79.9±3.0 |  | 75.4±4.7 | 83.6±4.4 | 77.7±4.6 |
| Height (% predicted) | 97.4 ±1.0 | 95.2±1.2 |  | 96.0±3.9 | 96.6± 1.8 | 95.5±1.3 |
| Weight/Height (% predicted) | 86.7±2.1 | 86.2± 1.9 |  | 80.3±4.8 | 89.7±2.8 | 84.0±3.2 |
| *P. aeruginosa* infection | 63.4 % | 68.6 % |  | 75.0 % | 63.6 % | 64.2 % |
