# Supplementary material for: TNF gene polymorphisms in cystic fibrosis patients: contribution to the disease progression
Source: J Transl Med. 2013 Jan 23;11:19. doi: 10.1186/1479-5876-11-19 (PMC3565881; doi:10.1186/1479-5876-11-19)
Supplement: Additional file 2 — Table S2. Forced vital capacity (FVC and FEV1) in CF patients with different TNF genotypes. [file 1479-5876-11-19-S2.docx]

**Table S2**  Frequencies of various TNF genotypes among CF patients and Healthy subjects

| *TNF* genes |  | Healthy control | CF patients | P |
| --- | --- | --- | --- | --- |
| TNF-α-308G/A – LT-α+252A/A |  | 6/127 (4.7 %) | 13/190 (6.8 %) | 0.290 |
| TNF-α-308G/G – LT-α+252A/A |  | 67/127 (52.8 %) | 98/190 (51.6 %) | 0.460 |
| TNF-α-308G/A – LT-α+252A/G |  | 18/127 (14.2 %) | 30/190 (15.8 %) | 0.411 |
| TNF-α-308G/G – LT-α+252A/G |  | 21/127 (16.5 %) | 37/190 (19.5 %) | 0.305 |
| TNF-α-308G/G – LT-α+252G/G |  | 3/127 (2.4 %) | 4/190 (2.1 %) | 0.661 |
| TNF-α-308G/A – LT-α+252G/G |  | 9/127 (7.1 %) | 8/190 (4.2 %) | 0.194 |
| TNF-α-308A/A – LT-α+252A/G |  | 2/127 (1.6 %) | Not found | 0.160 |
| TNF-α-308A/A – LT-α+252G/G |  | 1/127 (0.8 %) | Not found | 0.401 |
