# Supplementary material for: TNF gene polymorphisms in cystic fibrosis patients: contribution to the disease progression
Source: J Transl Med. 2013 Jan 23;11:19. doi: 10.1186/1479-5876-11-19 (PMC3565881; doi:10.1186/1479-5876-11-19)
Supplement: Additional file 3 — Table S3. Frequencies of various TNF genotypes among CF patients and Healthy subjects. [file 1479-5876-11-19-S3.docx]

**Table S3**  Forced vital capacity (FVC and FEV_1_) in CF patients with different TNF genotypes

| Genotype | FVC | FEV_1_ |
| --- | --- | --- |
| *TNF-α–308GG* | 77.7 ± 2.1 (n = 136) | 69.9 ± 2.3 (n = 135) |
| *TNF-α–308GA* | 78.1 ± 3.7 (n = 49) | 72.1 ± 4.5 (n = 49) |
| *LT-α+252GG* | 76.1 ± 6.8 (n = 11) | 69.3 ± 8.9 (n = 11) |
| *LT-α+252GA* | 75.6 ± 2.8 (n = 60) | 69.9 ± 3.5 (n = 59) |
| *LT-α+252AA* | 80.8 ± 2.6 (n = 105) | 72.6 ± 2.8 (n = 105) |

All p ≥ 0.7
