# Supplementary material for: TNF gene polymorphisms in cystic fibrosis patients: contribution to the disease progression
Source: J Transl Med. 2013 Jan 23;11:19. doi: 10.1186/1479-5876-11-19 (PMC3565881; doi:10.1186/1479-5876-11-19)
Supplement: Additional file 4 — Table S4. Contribution of individual TNF-α and LT-α gene polymorphisms in CF associated cirrhosis with portal hypertension. [file 1479-5876-11-19-S4.docx]

**Table S4.** Contribution of individual *TNF-α* and *LT-α* gene polymorphisms in CF associated cirrhosis with portal hypertension

| *TNF* genes | Cirrhosis  (with PH) | *p*, p*** |
| --- | --- | --- |
| *TNF-α-308G/A – LT-α+252A/A* | 1/13 (7.7%) | >0.01 |
| *TNF-α-308G/G – LT-α+252A/A* | 6/97 (6.2%) | >0.01 |
| *TNF-α-308G/A – LT-α+252A/G* | 4/29 (13.8%) | >0.01 |
| *TNF-α-308G/G – LT-α+252A/G* | 1/37 (2.7%) | >0.01 |
| *TNF-α-308G/G – LT-α+252G/G*  *together with*  *TNF-α-308G/A – LT-α+252G/G* | 2/12 (16.7%) | >0.01 |

PH, portal hypertension

*** comparison with *TNF-α-308G/G – LT-α+252A/A*

**** comparison with *TNF-α-308G/A – LT-α+252A/G*
